# Nine SARS-CoV-2 Positive Pregnant Women and Their Infant Delivery Outcomes

**DOI:** 10.7759/cureus.11946

**Published:** 2020-12-07

**Authors:** Julia Fashner, Cristobal Cintron

**Affiliations:** 1 Family Medicine, University of Central Florida/HCA Healthcare GME Consortium, Ocala Regional Medical Center, Ocala, USA; 2 Epidemiology and Public Health, University of Central Florida/HCA Healthcare GME Consortium, Ocala Regional Medical Center, Ocala, USA

**Keywords:** covid-19, sars-cov-2, pregnancy, infant

## Abstract

There are scientific reports from around the world describing the cases of COVID-19. This is a case series reporting outcomes of deliveries from nine mothers with positive SARS-CoV-2 testing at Healthcare Corporation of America hospitals in the United States from January to April 2020. Thirty-three percent of the women had cesarean sections. There was only one preterm birth and that infant did have low birth weight and low Apgar scores at one and five minutes. Seven of the nine infants were tested for SARS-CoV-2 and all results were negative. As the COVID-19 pandemic continues across the globe and at a high rate in the United States, more research will be needed to determine the outcomes for pregnant women and their offspring, both at birth and in the future.

## Introduction

In the last 18 years, there have been three coronavirus outbreaks: severe acute respiratory system (SARS), Middle East respiratory syndrome (MERS), and now the COVID-19 pandemic. The scientific literature has been exploding with case reports and studies of patients infected with the 2019 novel coronavirus (SARS-CoV-2). The most vulnerable individuals for COVID-19 appear to be the elderly, but all ages can be infected with the virus. In considering other populations at risk, family physicians often regard a pregnant woman and her baby as a unit. 

Pregnancy is a unique life situation when there are two patients in a single body. This relationship creates unique immunologic issues during pregnancy [1]. The mother must fight off any illnesses, but not attack a baby which is not the same as her. Bacteria and viruses are a threat to both mom and baby, so women are routinely screened for some of these diseases during pregnancy (HIV, hepatitis B, chlamydia, gonorrhea, and group B strep), but some viruses are not routinely screened (cytomegalovirus and herpes simplex) [2]. From previous coronaviruses outbreaks (SARS and MERS), the pregnancy outcomes including higher rates of spontaneous abortion, premature birth, but no vertical transmission [3].

## Materials and methods

We queried the de-identified claims-based HCA Healthcare electronic data repository paid as of April 29, 2020, for pregnant women admitted in the U.S. (N=519). We selected women who had testing results for SARS-CoV-2 which were positive (N=9). The COVID testing was done on the day of admission up to three days into the patients stay. We collected the mother and infant clinical data for variables of interest. This study was approved by the HCA Healthcare IRB.

## Results

The collected data for the mother-baby pairs are reported in Table 1. The mean age (± 1 standard deviation (SD)) of the mothers was 28.2 ± 7 years old and the mean gestational age at the time of delivery was in the 37th week. The majority of the women were Hispanic (66%). Cesarean sections (C/S) were performed on three of nine of the women; one was preterm due to COVID disease. The length of stay varied from one to nine days, with a mean of 4.4 days. For the infants, the mean birth weight (± 1 SD) was 3119 ± 570 grams (one infant was low birth weight), with average Apgar at one minute of 7.3 ± 2.2 and average Apgar at five minutes of 8.1 ± 1.8. Four infants went to the newborn intensive care unit, likely due to their COVID exposure. Seven of the nine infants were tested for SARS-CoV-2 and all results were negative. 

**Table 1:** Maternal and neonatal characteristics for hospital tested COVID-19 positive mothers. y=year; wks=weeks; d=day(s); g=gram; n/a=not applicable; A=Asian; O=Other; W=White; H=Hispanic or Latino; N=not Hispanic or Latino; C/S: cesarean section; rC/S= repeat cesarean section; V=vaginal; LOS=length of stay; F=female; M=male; NEG=negative or not detected; NT=not tested; new=newborn nursery; NICU=newborn intensive care unit

	Case								
Characteristic	1	2	3	4	5	6	7	8	9
Age (y)	19	22	36	22	33	39	30	23	30
Race	O	W	W	W	W	A	W	W	O
Ethnicity	H	H	H	N	H	N	N	H	H
Gestational age (wks)	39	40	39	38	38	39	39	29	37
Delivery type	V	C/S	rC/S	V	V	V	V	C/S	V
Mother LOS (d)	2	4	3	6	1	1	1	9	4
Infant gender	F	M	M	F	F	F	F	M	F
Infant weight (g)	2869	3371	3820	3260	2900	3308	3325	1797	3419
Low birth weight	No	No	No	No	No	No	No	Yes	No
Preterm	No	No	No	No	No	No	No	Yes	No
Infant Apgar at 1 minute	8	3	8	9	9	9	8	4	8
Infant Apgar at 5 minutes	9	5	9	9	9	9	9	5	9
Infant COVID test (days from admission)	1	n/a	n/a	3	1	1 and 2	1	1	1
Infant COVID test result	NEG	NT	NT	NEG	NEG	NEG	NEG	NEG	NEG
Infant location	New	NICU	New	NICU	New	New	New	NICU	NICU
Infant LOS (d)	2	4	3	3	4	2	3	35	2

## Discussion

In the current literature, several systematic reviews of COVID-19 pregnancy cases have shown preterm birth is common with low birth weight and a high rate of cesarean section (mostly in the Chinese studies) [4-8]. The Centers for Disease Control in the US reported the 2017 and 2018 rate of C/S was about 32% [9]. Della Gatta et al. included six studies with a total of 48 deliveries, 46 of which were by C/S [4]. They report the median maternal age was 30 years and median gestation was 36.5 weeks (preterm is less than 37 weeks). Our patients were slightly younger and only one woman had a preterm delivery. They also reported nine patients had premature rupture of membranes. Of the infants born, there were two deaths and no vertical transmission of SARS-CoV-2 in any tested infant. 

Lamouroux et al. included 12 articles with 68 deliveries, 64 of which were by C/S [5]. Multiple specimens were tested for SARS-CoV-2 including amniotic fluid, placenta, cord blood, vaginal swab, and breast milk, none of which were positive. They report one infant born by C/S who tested positive on a pharyngeal swab, but this newborn had no contact with the mother after birth. The authors do not conclude if this was a vertical transmission. Panahi et al. included 13 articles with 37 pregnant mothers between age 23 and 40 years old [6]. In this group, 29 women had C/S; six had preterm labor and six had premature ROM. All infants had Apgars of 8-10, but one infant did die during the neonatal period. For our report, seven of the nine infants had Apgars score of 8-9 and the mothers' ages were 19-39 years old, yet only one of the infants was born preterm.

Trocada et al. included eight studies with 51 deliveries, 48 of the infants were tested and all results were negative [7]. Delivery was mostly by C/S (94%) and full-term (65%). Birth weights were available for 40 neonates with a mean of 2292 grams (20% were low birth weight). The infants in our series all tested negative but had a higher mean birth weight, likely because there were more term births. Zaigham et al. had 18 studies with 86 deliveries (92% were by C/S) in women age 29 to 32 years old [8], a much narrower age range than our report. Our group has only one preterm birth. Each of the included studies did not contain all the same information, so they report premature delivery occurred in 20 of 48 reported cases. Of course, because COVID-19 is an emerging disease with limited cases published, there is overlap for included studies among these systematic reviews. 

There are many challenges in caring for pregnant women who may get infected with SARS-CoV-2, along with the risk that the infant is exposed to the virus in utero or after birth. The guidance was given for infection control due to the experiences in SARS and MERS [10,11]. The International Society of Infectious Disease in Obstetrics and Gynecology (ISIDOG) guideline recommends pregnant women to take extensive preventive measures like disinfecting surfaces with >60% ethanol and strict social distancing. They also advise if the pregnant woman worked in high-risk areas (respiratory therapists, intensive care units, etc.) they should transfer to low-risk areas. While women may not be more susceptible to infection with SARS-CoV-2 [10], the ISIDOG considers pregnant women high-risk due to changes in the immunologic system that make them at increased susceptibility to pathogens [11]. 

Management for neonates born to COVID-19 mothers include decisions about who attends the delivery, whether to separate the infant from mom to decrease exposure to SARS-CoV-2 and isolating them from other neonates not born to COVID-19 mothers. At this time, the Centers for Disease Control believe transmission of SARS-CoV-2 to infants is from postnatal exposure to their mother or other caregivers with the virus and not vertical transmission due to the limited evidence [12]. Separating the infant and mother should involve shared medical decision making and this discussion should occur before delivery. 

Physicians will need to keep in mind the inflammatory state due to COVID-19 may cause later effects in the child [1]. Maternal inflammation and immune activation have been linked to neuro-development changes leading to attention deficit hyperactivity disorder and autism-like syndrome [1]. As the COVID-19 pandemic continues across the globe and at a high rate in the United States, analyzing the research will help guide the care of pregnant women and their children, both at birth and in the future.

There are several limitations to our report. We are not able to present all pertinent clinical data for the moms and babies as we did not review all their medical records. The material available to us is from the claims database. This sample may not represent all COVID-19 mothers treated at HCA Healthcare facilities, as we included SARS-CoV-2 tests available in the claims data only and not from other labs. The type of SARS-CoV-2 test is also not known because this is not in the claims database. The reliability of different tests has been disputed during the pandemic.

## Conclusions

Physicians who care for pregnant women who get infected with SARS-CoV-2 face many challenges. They know there is a risk for mother and infant exposed to the virus in utero or after birth. Pediatricians, neonatologists, and other physicians caring for infants also recognize the difficulties this novel coronavirus has imposed on the treatment of newborns exposed to the virus. As more data is published, the scientific community will be able to analyze the findings and consider protocols for the care of pregnant women and their infants exposed to COVID-19. Decisions regarding what type of delivery (vaginal or cesarean section), screening all pregnant women admitted for labor, and quarantine of the infant if the mother tests positive will be aided by more case study reports and research literature. 
